# Giant coronary artery aneurysm in infantile Kawasaki disease: When to use cardiac computed tomography angiography

**DOI:** 10.1002/ccr3.3227

**Published:** 2020-09-19

**Authors:** Shreeja Kadakia, Chi Chi Do‐Nguyen, Maxwell F. Kilcoyne, Randy M. Stevens, Erika Lindholm, Autumn Nanassy, James Starc, Mary G. Mallon

**Affiliations:** ^1^ Drexel University College of Medicine Philadelphia Pennsylvania; ^2^ Philadelphia College of Osteopathic Medicine Philadelphia Pennsylvania; ^3^ St. Christopher’s Hospital for Children Philadelphia Pennsylvania

**Keywords:** cardiac computed tomography angiography, coronary artery aneurysm, infant, Kawasaki disease, ultrasound

## Abstract

Transthoracic echocardiography is the imaging modality of choice for the detection of coronary artery aneurysms (CAAs) in Kawasaki disease. However, cardiac computed tomography angiography is useful in the diagnosis of distal CAAs.

## Graphical abstract

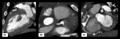

## 1 INTRODUCTION

Transthoracic echocardiography is used to detect coronary artery aneurysms (CAA) in Kawasaki disease, but diagnosing distal CAAs is challenging. Computed tomography angiography (CTA) in a 6‐month‐old female with four CAAs revealed two additional areas of dilation. Cardiac CTA is useful in the diagnosis of distal CAAs associated with Kawasaki disease.

Kawasaki disease (KD) is an immune‐mediated vasculitis that has a predilection for medium‐sized arteries, especially the coronary arteries. It typically occurs in children between 6 months and 5 years of age and is the major cause of acquired pediatric coronary artery disease; yet, timely identification of coronary artery aneurysms (CAAs) can be an immense clinical challenge. Currently, transthoracic echocardiogram (TTE) is the diagnostic imaging modality of choice to screen for CAAs.^1^ Despite its widespread use, TTE is inadequate in visualizing distal vasculature.^2^, ^3^ Development of CAAs can happen within a week of disease onset and lead to myocardial infarction, chronic ischemia, and sudden death. In addition, these patients have an increased risk of developing coronary artery disease later in life and thus, a well‐timed and appropriate workup is crucial.^4^ Several other modalities, including cardiac computed tomographic angiography (CTA), have been useful in evaluating the severity of coronary artery abnormalities, particularly in the distal coronary vasculature.^1^ This report demonstrates the potential benefits of using CTA in patients with known CAA secondary to KD.

## 2 CASE REPORT

A 6‐month‐old female was admitted to an outside institution, after presenting to the emergency department with a 2‐week history of rash on her hands and feet, eye redness, and fever. After being diagnosed with KD, she received two rounds of intravenous immunoglobulin (IVIG), Remicade, a steroid taper, and started on aspirin therapy. This treatment strategy was employed to suppress inflammation as much as possible due to evidence of ongoing inflammation and remodeling of her coronary arteries after one dose of IVIG and to prevent further damage. At the time of her discharge, a TTE demonstrated mild dilation of the right coronary artery (RCA). Two weeks after discharge, she presented for follow‐up TTE which demonstrated an increase in RCA (3.3 mm, z‐score: 8.0), left main coronary artery (LMCA) (2.3 mm, z‐score: 2.4), and left anterior descending (LAD) coronary artery aneurysm size (2.0 mm, z‐score: 4.6). This prompted the addition of Plavix to her regimen, since she met the criteria for dual‐antiplatelet therapy. A follow‐up TTE revealed alarming new findings: a giant aneurysm of the proximal RCA (5.0 mm, z‐score + 11) and dilation and aneurysms of the LMCA (3.3 mm, z‐score: 3.7), proximal LAD (2.6 mm, z‐score: 4.9), and left circumflex artery (LCX 2.4 mm). Due to the number and sizes of the CAAs, the patient was admitted to the hospital for additional imaging and initiation of anticoagulation. Upon admission, physical examination showed a well‐appearing infant; however, laboratory results indicated inflammation and thrombocytosis, as shown in Table 1. The patient was hemodynamically stable and received therapeutic dosing of Lovenox and intravenous Solumedrol therapy.

**Table 1:** Patient's abnormal laboratory values following admission, demonstrating inflammation and thrombocytosis

Erythrocyte sedimentation rate (ESR)	105 mm/h (normal: 3‐13 mm/h)
Platelet count	528 000/mL (normal: 150 000 and 400 000/mL)
C‐reactive protein (CRP)	7.6 mg/L (normal: 2‐5 mg/L)

**FIGURE 1:**
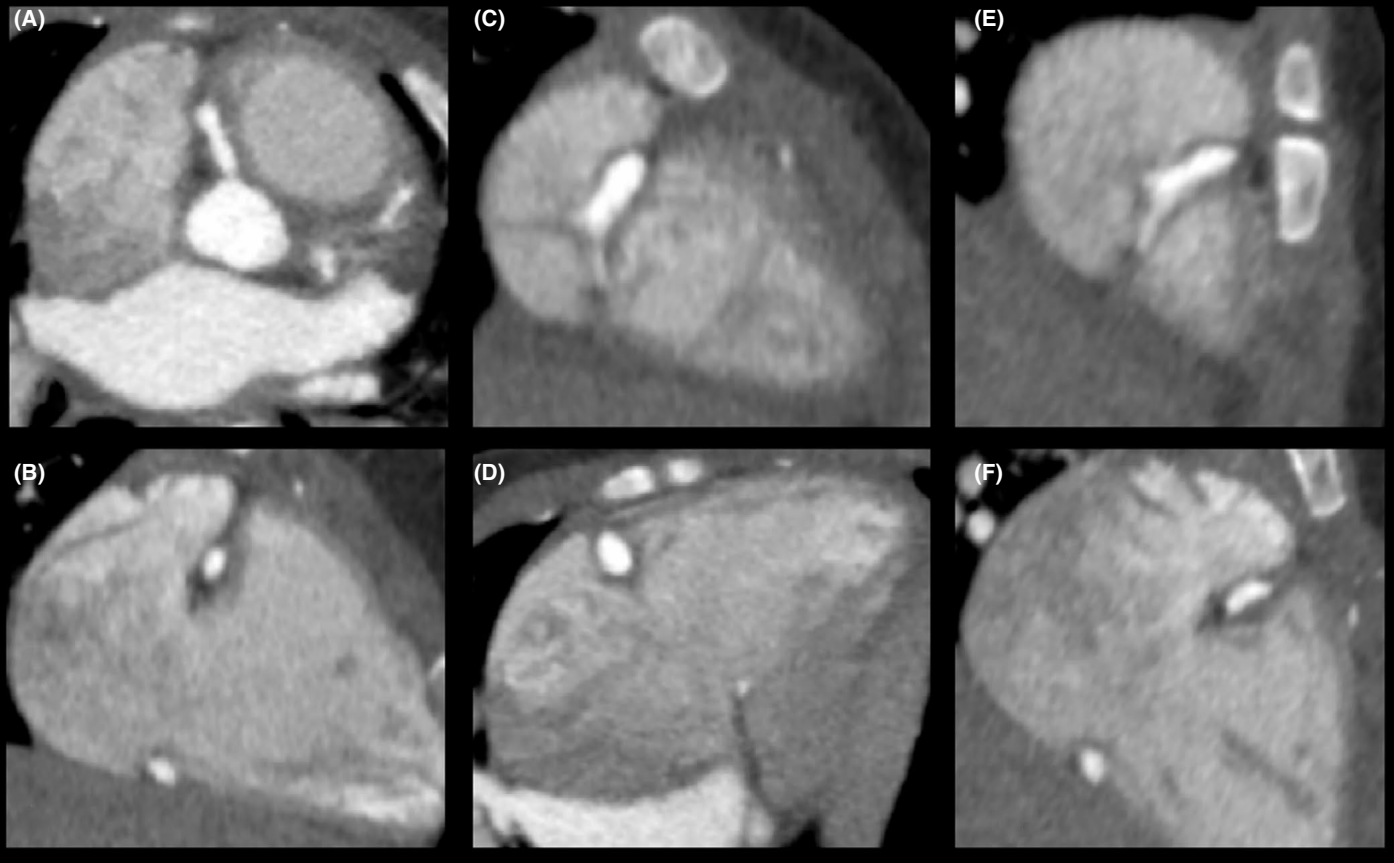
Contrast‐enhanced CT angiographic images of the RCA, demonstrating three regions of aneurysmal dilatation. Proximal RCA with aneurysmal dilatation in the perpendicular short‐axis view (A) and in the long‐axis view (B), in total measuring 4.3 × 2.9 × 9.8 mm. The short axis (C) and the long axis (D) of the aneurysmal dilatation of the RCA at the origin of the acute marginal artery, measuring 6.4 × 4.8 × 10 mm. Likewise, in the short axis (E) and the long axis (F), it is depicted at the bifurcation of the PDA and the atrioventricular nodal artery, measuring 4.5 × 3.2 × 11 mm

**FIGURE 2:**
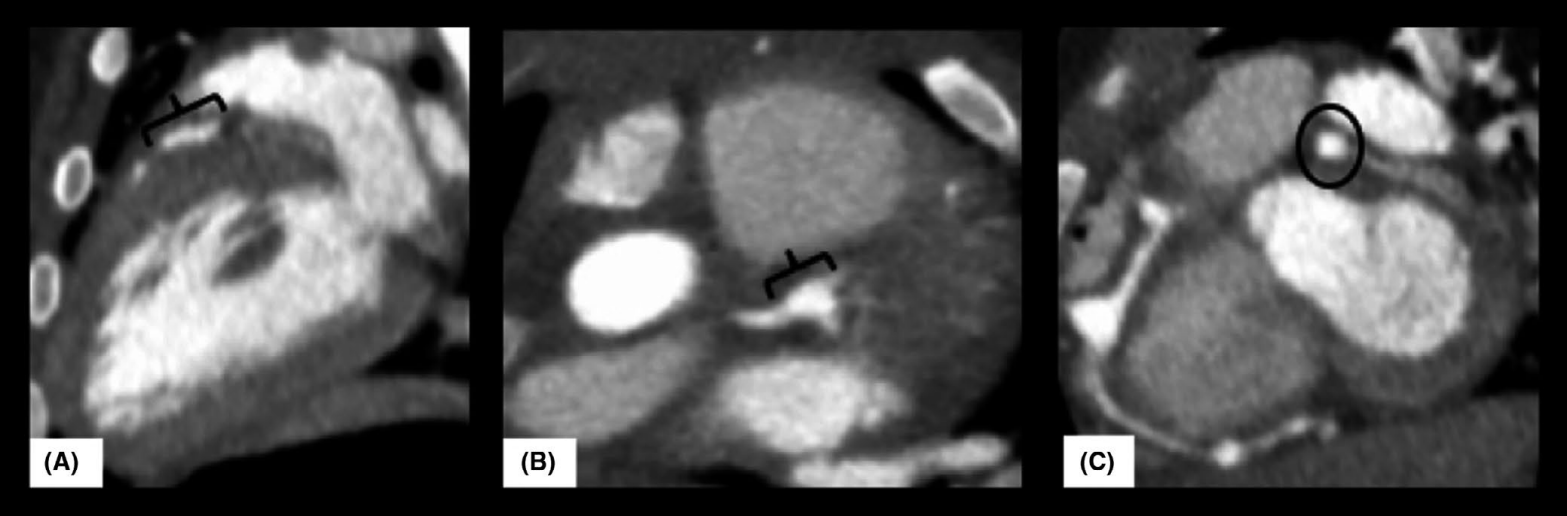
Contrast‐enhanced CT angiographic images of the LMCA and branches. Image A demonstrates a prominent LAD with irregular margins without aneurysmal dilatation of the segment between the bifurcation with the LCX and the first septal branch. Image B in long axis ({) and image C in short axis (○) depict aneurysmal dilatation of the bifurcation of the LAD and LCX, measuring 4.3 × 4.0 × 5.1 mm

The patient underwent CTA of the coronary arteries, which demonstrated three regions of aneurysmal dilation of the RCA: a portion of the conal branch, acute marginal branch, and bifurcation of the posterior descending (PDA) and atrioventricular (AV) nodal branches (Figure 1). There was also aneurysmal dilation at the bifurcation of the LAD and LCX and irregular margination of the LAD (Figure 2). The admission TTE and CTA findings are summarized in Table 2.

**Table 2:** Comparison of pre‐admission TTE findings and CTA findings

TTE Findings	CTA Findings
RCA Aneurysm (Diameter: 5 mm, z‐score: +11)	Three RCA Aneurysms Conal Branch region 4.3 × 2.9 × 9.8 mm Acute Marginal artery region 6.8 × 4.8 × 10 mm Bifurcation of the PDA and AV nodal artery 4.5 × 3.2 × 11 mm
Moderate aneurysms of the LMCA, prox. LAD, and prox. LCX	Aneurysm at bifurcation of the LAD and LCX 4.3 × 4 mm
	Additional Findings: Prominent LAD without frank aneurysm near the bifurcation of the LCX and origin of the first septal branch

Given her extensive CAA, she was discharged on low‐dose prednisolone taper for 4 months. The patient has remained on 81 mg of PO aspirin daily and given the giant coronary aneurysms, she most likely will for life. Serial echocardiograms demonstrated continuous improvement of the CAA. A CTA at 18 months after diagnosis, the proximal RCA is mildly dilated (2.9 × 2.6 mm) and left coronary system size is within normal limits. She has remained asymptomatic but is considered high risk for coronary artery disease and will undergo continued lipid screening and exercise stress tests to monitor her coronary disease throughout her life.

## 3 DISCUSSION

As seen with our patient, CAA can occur despite proper IVIG and aspirin therapy with a reported incidence of is 4%‐6%. While 90% of CAAs resolve within 2 years, the seriousness of potential sequela makes serial imaging key to assessing disease progression and guiding management.^4^ TTE is the imaging modality of choice for screening for CAAs in KD, with a high sensitivity for detecting coronary lesions in the proximal RCA and LMCA; however, the sensitivity decreases in the more distal portions of the arteries.^5^ There is currently no consensus regarding when TTE should be supplemented with CTA to investigate coronary artery involvement, but as seen in our case and other retrospective series, CTA warrants utilization when managing patients at risk for CAAs.^5^, ^6^

Accurate imaging of CAAs is crucial because z‐score is the most powerful predictive factor for coronary events.^4^, ^7^, ^8^ In a retrospective analysis of 1356 patients, the incidence of coronary artery events (thrombosis, stenosis, intervention, MI, and death) with CAA with z‐scores > 10 and absolute diameter of >8 mm was 48% while those with z‐scores < 10 and absolute diameter < 8 mm had an incidence of 1%.^7^ This dramatic difference in morbidity and mortality is the reason why management is heavily based on imaging findings. While patients with small CAAs (z‐score: 2‐5) are treated with low‐dose aspirin, those with giant CAAs (z‐score > 10), as seen in our patient, are started on systemic anticoagulation, such as low molecular weight heparin, and an antiplatelet agent until coronary dimensions improve.^4^ In our case, one giant CCAs of the RCA was detected by TTE, which led to the patient getting a CTA. The fact that the two giant distal RCA aneurysms were missed by TTE means if the proximal CCA was not present, then these aneurysms would have gone completely undetected. However, it is important to note that distal CAA in the absence of proximal CAA is very rare. In a retrospective series of 25 patients undergoing both TTE and CTA, CTA was consistently superior in identifying CAAs. Twenty‐three lesions were missed on TTE and found on CTA with 66% of them being distal lesions. In addition, CTA was also able to provide a more detailed and accurate description on size, shape, and presence of stenosis or thrombosis of the CAAs, which has been progressively more important in risk stratification.^5^, ^8^, ^9^

While the sensitivity of TTE is not as high as CTA, TTE can evaluate the distal coronary artery anatomy well in young children with good echocardiographic windows and sedation when necessary. Therefore, CTA is not necessary for all patients diagnosed with KD. However, those who are at high risk for coronary lesions may benefit from CTA being involved earlier in their management.^6^ In a retrospective series, Friedman et al analyzed 500 KD patients with CAA and found TTE showing involvement of both left and right coronary arteries, z‐score > 5 for any aneurysm, or lack of IVIG treatment as predictors of progressive disease.^4^ While Chen et al found CAAs smaller than 5.6 mm, no calcification, and ectatic shape were more likely to regress.^8^ In addition to initial TTE findings, Son et al found an age < 6 months, Asian race, and a CRP of 13 mg/dL or higher are all significant predictors of CAAs.^10^ Utilizing factors like initial TTE findings, laboratory markers, and demographic data is a promising way to create a scoring nomogram to help clinicians elucidate who would benefit from additional imaging with CTA. This is may be a future project to further evaluate which cases benefit from additive information from CTA and when in the disease course a CTA should be considered.

While the American Heart Association has included CTA as a reasonable imaging modality in patients where TTE is felt to be inadequate, there is some hesitation in utilizing CTA in the pediatric population.^11^ Previous reports have argued that an asymptomatic patient should not have a CTA done due to the risk of IV contrast administration and radiation exposure. These concerns are because physiologically elevated heart rates in children have been associated with an increase in sensitivity to radiation.^6^ Our patient is an example that lack of symptoms does not preclude advancing coronary lesions that warrant investigation.^5^ The findings from the CTA are what changed management to include more frequent post‐discharge TTEs, follow‐up CTA, an extended steroid regimen, and life‐long aspirin use. A teaching point of this case is that patients with CA enlargement early in the course or IVIG resistance need close follow‐up. In this case, 2 weeks elapsed from hospital discharge to a follow‐up echo. This should have occurred much earlier. In addition, there are ways to safely overcome the radiation exposure; it was found that a dual‐source coronary CTA with a beta blocker protocol can decrease the radiation dosage in children to 1/3‐1/5 the dosage found in invasive angiography.^6^ Not to mention, coronary CTA eliminates the risk of procedural complications associated with invasive angiography and does not require hospitalization. Along with being time efficient, it is non‐invasive and allows for appropriate evaluation of the distal coronary vasculature.^12^

## CONFLICT OF INTEREST

None.

## AUTHOR CONTRIBUTIONS

SK: extracted patient history and vital imaging data from the medical records, drafted manuscript, and assisted in completing edits. CCDN: extracted patient history and vital imaging data from the medical records, drafted manuscript, assisted in completing edits, and submitted the manuscript. MFK: extracted patient history and vital imaging data from the medical records, drafted manuscript, and assisted in completing edits. RMS: helped with the conceptual design of the manuscript, provided oversight to all aspects of the manuscript, and contributed with his expertise and skill in the field. EL: helped with the conceptual design of the manuscript, provided oversight to all aspects of the manuscript, and contributed with her expertise and skill in the field. AN: helped with the conceptual design of the manuscript, provided oversight to all aspects of the manuscript, and contributed with her expertise and skill in the field. JS: helped with the conceptual design of the manuscript, provided oversight to all aspects of the manuscript, and contributed with his expertise and skill in the field. MGM: helped with the conceptual design of the manuscript, provided oversight to all aspects of the manuscript, and contributed with her expertise and skill in the field.
